# Downregulated CCND3 Is a Key Event Driving Lung Adenocarcinoma Metastasis during Acquired Cisplatin Resistance

**DOI:** 10.7150/ijbs.100921

**Published:** 2025-01-01

**Authors:** Yun Su, Yuting Ma, Yubing Wang, Ping Xu, Miaoling Guo, Haolin Cao, Jianyang Xin, Xi Wu, Xiaoyan Liu, Shan Chen, Xingyu Tao, Huiling Yang, Chao Cheng, Rongquan Huang, Rongshuai Pan, Yuexin Pan, Beixian Zhou, Weiyi Fang, Zhen Liu

**Affiliations:** 1 Guangzhou Municipal and Guangdong Provincial Key Laboratory of Protein Modification and Degradation, Guangzhou Medical University, Guangzhou 511436, China.; 2 Department of Cardiothoracic Vascular Surgery, Guangzhou Eighth People's Hospital, Guangzhou Medical University, Guangzhou 510440, China.; 3 Department of Pulmonary and Critical Care Medicine Peking University Shenzhen Hospital, Shenzhen 518036, China.; 4 School of Pharmacy, Guangdong Medical University, Dongguan 523808, China.; 5 Department of Otolaryngology, Shenzhen Longgang Otolaryngology Hospital, Shenzhen 518172, China.; 6 The People's Hospital of Gaozhou, Gaozhou 525200, China.; 7 Cancer Center, Integrated Hospital of Traditional Chinese Medicine, Southern Medical University, Guangzhou 510315, China.; 8 Department of Pathology, Guangzhou Municipal and Guangdong Provincial Key Laboratory of Protein Modification and Degradation, School of Basic Medical Sciences, Guangzhou Medical University, Guangzhou 511436, China.

**Keywords:** lung adenocarcinoma, CCND3, vimentin, metastasis, acquired DDP chemoresistance

## Abstract

Cyclin D3 (CCND3), a member of the cyclin D family, is known to promote cell cycle transition. In this study, we found that CCND3 was downregulated in cisplatin-resistant (*cis*-diamminedichloroplatinum, DDP) lung adenocarcinoma (LUAD) cells. The loss of CCND3 indeed impeded cell cycle transition. Unexpectedly, its downregulation significantly triggered cytoskeleton remodeling and chemoresistance and accelerated LUAD metastasis *in vivo* and *in vitro*. Moreover, the clinical samples showed a significant negative correlation between CCND3 expression and lymphatic metastasis, as well as the unfavorable survival prognosis of patients with LUAD. Mechanistically, CCND3 downregulation in DDP-resistant LUAD cells was attributable to the transcriptional suppression of PI3K/Akt/c-Jun signaling. Reduced CCND3 expression diminished the recruitment of the E3 ubiquitin ligase PARK2 to ubiquitinate and degrade the vimentin protein, thus triggering epithelial-mesenchymal transition (EMT) to result in cytoskeleton remodeling-stimulated metastasis and chemotherapeutic resistance in LUAD. These results demonstrated that activated PI3K/Akt/c-Jun significantly suppressed CCND3 expression, thereby inhibiting vimentin degradation via PARK2-mediated ubiquitination in DDP-resistant LUAD cells. This, in turn, promoted EMT, facilitating cytoskeleton remodeling-stimulated metastasis and chemoresistance to DDP. Overall, these findings provided a new perspective on the role of CCND3 in LUAD progression and acquired cisplatin resistance.

## Introduction

Lung adenocarcinoma (LUAD) represents the most common subtype of lung cancer, accounting for approximately 40% of all lung cancer cases 1. Unfortunately, the overall survival of patients with advanced LUAD remains less than 5 years 2, primarily due to the persistent challenges of its metastasis and therapeutic resistance 3. Therefore, improving our understanding of the mechanisms of metastasis and chemotherapeutic resistance in LUAD may help in improving the survival and prognosis of patients.

Epithelial-mesenchymal transition (EMT) is a process of cell remodeling in which the epithelial cells acquire mesenchymal phenotypes with enhanced cell motility 4. The activation of EMT is considered to contribute to tumor initiation, metastasis, and resistance to chemotherapy in cancer 5, 6. Cisplatin (cis-diamminedichloroplatinum, DDP) is the standard first-line chemotherapeutic agent used in patients with advanced LUAD. However, unfortunately, its clinical use is frequently hampered by intrinsic and acquired drug resistance during chemotherapy. Among the various reported mechanisms, the activation of EMT has been identified as one of the major contributors to tumor cell resistance to DDP. On the other hand, accumulating evidence shows that DDP-resistant cancer cells exhibit EMT and enhanced metastatic potential 7-9. Nevertheless, studies on how DDP-associated acquired drug resistance promotes tumor metastasis via EMT are still scarce, and therefore the underlying molecular mechanism needs to be urgently explored.

In this study, we established DDP-resistant LUAD cell lines. We found that the cell cycle of the resistant cell models was restrained with the induction of DDP in the G2/M phase, concomitant with a weaker proliferation capacity compared with parental cells. In contrast, the invasive property of these cell models was enhanced. More prominently, the resistant LUAD cells not only acquired an EMT-like phenotype with alteration from the epithelial to mesenchymal morphology but also underwent a shift in cytoskeleton remodeling. This phenomenon was further validated by the observed changes in cell cycle-associated proteins and EMT biomarkers. The expression of cyclin family proteins, especially cyclin D3 (CCND3), was significantly downregulated in DDP-resistant LUAD cells, whereas the expression of the cytoskeletal protein vimentin was upregulated.

The cyclin family, specifically cyclin D proteins (cyclin D1, D2, and D3 subtypes), is a positive regulator of the G1 phase. Similar to other D cyclins, CCND3 is overexpressed in human cancers and serves as a potential prognostic biomarker 10, 11. Most studies have focused on the role of CCND3 in stimulating cell cycle transition and inducing cell proliferation in tumors 12-15. However, the role and potential molecular mechanisms of CCND3 in DDP resistance and tumor metastasis remain less explored. Han *et al.* found that miR-138 inhibited the proliferation, migration, and invasion of non-small-cell lung cancer cells and increased the sensitivity to chemotherapy by directly targeting CCND3 16, indicating that a reduction in CCND3 increases the sensitivity of non-small-cell lung cancer cells to DDP. Another study showed that CCND3, which was transcriptionally regulated by ATF5, increased DDP-induced apoptosis in HeLa cells 17, suggesting that upregulated CCND3 sensitized HeLa cells to DDP. The data presented in this study contradicted Han's results, thus indicating the degree of complexity regarding CCND3 in DDP resistance. However, the role of CCND3 in DDP resistance and LUAD metastasis, as well as the detailed mechanism involved, remain to be clarified.

The present study found that the transcription of CCND3 was suppressed by PI3K/AKT-induced c-Jun, which was activated in DDP-resistant LUAD cells. Downregulated CCND3 reduced the PARK2 recruitment to ubiquitinate and degrade vimentin, which induced EMT to promote LUAD metastasis and acquired chemoresistance to DDP. Our findings were the first to reveal the role and mechanism of CCND3 in drug resistance and metastasis of LUAD, suggesting that CCND3 downregulation is not only a significant marker of acquired drug resistance but also a crucial factor underlying LUAD metastasis.

## Materials and Methods

### Cell lines and cell culture

A549 and PC9 cells were purchased from the Shanghai Cell Bank of the Chinese Academy of Sciences, China. DDP-resistant LUAD cells were constructed by treating the corresponding parental lines (A549 and PC9) with gradient DDP (Sigma, USA) concentrations. Cells were cultured in RPMI 1640 (Gibco, USA) with 10% fetal bovine serum (FBS, PAN, USA) and penicillin (100 U/mL)/streptomycin (100 U/mL) (Biosharp, China). To maintain the DDP-resistant phenotype of A549-DDP and PC9-DDP cells, DDP was added to the RPMI-1640 culture media (with a concentration of 6 μM).

### Western blot analysis and Immunofluorescence

Western blot analysis and immunofluorescence were performed as described in a previous study 18. Antibodies were listed in Table S1.

### Small interfering RNAs (siRNAs)

siRNAs targeting CCND3 and vimentin (VIM) were designed by RIBOBIO (Guangzhou, China). For transfection, siRNAs were complexed with Ribo FECTTMCP Transfection Kit (RIBOBIO, China) and transfected into A549 or PC9 cells following the manufacturer's descriptions. Target sequences of siRNAs were listed in Table S2.

### Lentivirus production

The establishment of lentiviral vectors harboring short hairpin RNA (shRNA) -targeting CCND3 and the construction of A549 and PC9 cell lines stably expressing shRNAs were performed as described in a previous study 19. shRNA sequences were designed targeting *CCND3* mRNA [GenBank: NM_001760] (Table S3). Lentivirus packaging and the production of lentiviral particles were provided by GeneChem Co., LTD (Shanghai, China).

### *In vivo* metastasis assay in nude mice

1.5×10^6^ A549-Lv-shCCND3-3 cells or A549-Lv-shNC Cells were intravenously inoculated into the tail veins of the 5-week-old female BALB/c nude mice (n = 5 per group). After six weeks, all mice were euthanized and the lungs were subjected to fluorescent image detection with LT-9MACIMSYSPLUS whole-body imaging system (Lighttools Research, Encintas, CA, USA). The ImageJ software (National Institutes of Health, USA) was used to quantify the fluorescence signal intensity. Lung metastases were confirmed by H&E staining.

This study was carried out in conformity with the regulations on animal research and ethics.

### Co-immunoprecipitation (Co-IP) and ubiquitination analyses

Co-IP was performed with the Pierce Co-Immunoprecipitation Kit (Thermo Scientific, USA) according to the manufacturer's instructions. Antibodies were listed in Table S1.

For ubiquitination analysis, cells were treated with 20 μM MG132 for 5 h before harvesting, and then performed Co-IP. Ubiquitin antibody was used to incubate on the corresponding membranes.

### Liquid chromatography-tandem mass spectrometry (LC-MS/MS)

A small portion of protein lysates from A549-DDP cells expressing flag-labeled CCND3 was set aside as a positive control, and the remaining lysates were equally divided into two halves respectively incubated with anti-IgG and anti-flag antibodies in Co-IP assays. After electrophoresis on SDS-PAGE gel, proteins from the Co-IP products and whole cell lysates were silver stained or Coomas bright stainied. All bands shown in the Co-IP product incubated with anti-IgG antibody were excluded as background signals. The differential bands between IgG group and IP group in polyacrylamide gel were compared longitudinally in the silver-stained gel to identify the specific bands formed by the CCND3-binding proteins. Meanwhile, according to the positions of differential silver-stained bands, protein bands of the IP group were excised from Coomassie stained polyacrylamide gel and sent for LC-MS/MS at Sangon Biotech Co., Ltd. (Shanghai, China). All the identificated proteins within the molecular weight range of 55-70 kDa (including vimentin) are listed in Table S7.

### Chromatin immunoprecipitation assay (ChIP)

DNA-protein complexes were immunoprecipitated from A549-DDP and PC9-DDP cells by the ChIP Kit (Thermo Scientific, USA) according to the manufacturer's protocol with c-Jun and Normal Rabbit IgG antibodies (Table S1). Precipitated DNA was subjected to qRT-PCR analysis using specific primers (Table S4) to amplify the promoter region of CCND3.

### Luciferase reporter assay

*CCND3* promoter sequences containing wild-type or mutant c-Jun binding sites were synthesized and constructed into the pGL3-Basic vector (named as CCND3-WT, CCND3-Mut1, CCND3-Mut2 and CCND3-Mut1+Mut2) by IGE Biotechnology LTD (Guangzhou, China). A549 and PC9 (5 × 10^5^) cells were seeded into 12-well plates one day before transfection. When the cells grew to about 70% density, c-Jun or control plasmids were transfected into the cells. After 24 h, Renilla vectors were co-transfected with CCND3 wide-type or mutant constructs under the condition of transfection. Luciferase activity was measured with Tecan Enzyme Calibrator (Hombrechtikon, Switzerland) 24 h after transfection. The luciferase activity was defined as the ratio of firefly luciferase activities versus Renilla luciferase activities.

### Tissue specimens and immunohistochemistry (IHC)

Tissue microarrays of LUAD (n=184) were purchased from Shanghai Outdo Biotech Co. LTD. (Shanghai, China). 29 paraffin-embedded primary LUAD specimens and 12 cases of metastatic lymphatic tissues were obtained from Peking University Shenzhen Hospital (Shenzhen, China). The clinicopathological features of all the 213 patients with LUAD are summarized in Table S5. All samples had a definite pathological diagnosis. The staining and evaluation of paraffin-embedded sections were performed as previously described 20. The score of each specimen was divided into two ranks: 0 to 3 stands for low expression, 4 to 7 stands for high expression. Two pathologists examined the stained tissue sections independently.

### Half-maximal inhibitory concentration assay

Half maximal inhibitory concentration (IC_50_) of cells was assessed using a Cell Counting Kit-8 (CCK-8) kit (Beyotime, China) according to the manufacturer's protocol. In brief, cells were seeded into 96-well plates at appropriate density. After cell attachment, medium containing an increasing concentration of DDP (0 μM, 1μM, 2 μM, 4 μM, 8 μM, 16 μM, 32 μM, and 64 μM) was added to the corresponding wells. Then the plate was incubated for 48 h. The optical density was measured at 490 nm with a microplate reader (BioTek, USA). All assays were performed in triplicate.

### EdU incorporation assay

Cell-Light EdU Apollo567 *In Vitro* Kit was purchased from RiboBio Corporation (Guangzhou, China) and was applied for the EdU incorporation assay. After culturing with EdU (50 μM) for 2 h, the cells were fixed with paraformaldehyde (4%), permeabilized with Triton X-100 (0.5%), and costained with DAPI and Apollo fluorescent dyes.

### Cell migration and invasion assay

2× 10^4^ cells in 200 µL RPMI-1640 without FBS were seeded in the upper chamber of 24-well chambers (BD Biosciences, USA) for migration assay or Matrigel Invasion Chamber (Corning, USA) for invasion assay. The lower chamber was filled with 600 µL RPMI-1640 supplemented with 10% FBS. After 24-48 h of incubation, cells on the upper chamber were wiped away, retaining the cells on the lower surface. The retained cells were then fixed using methanol and stained with crystal violet. Three fields of view were randomly photographed and counted under a microscope (Nikon, Japan).

### Statistical analysis

Statistical analysis was performed using SPSS Statistics 25 software (SPSS Inc, USA) and GraphPad Prism 8 software (GraphPad Software Inc., USA). The comparison between paired samples was analyzed by Student's t-test. One-way analysis of variance was used for multiple groups. The correlation between gene expression and clinicopathological features was confirmed by the chi-square test. The measurement data is expressed in mean± SEM. *p*<0.05 was considered as statistically significant (*p<0.05, ** *p*<0.01, *** *p*<0.001, **** *p*<0.0001).

## Results

### DDP-resistant LUAD cells exhibited an EMT-like phenotype and a weaker proliferative capacity

**Figure 1:**
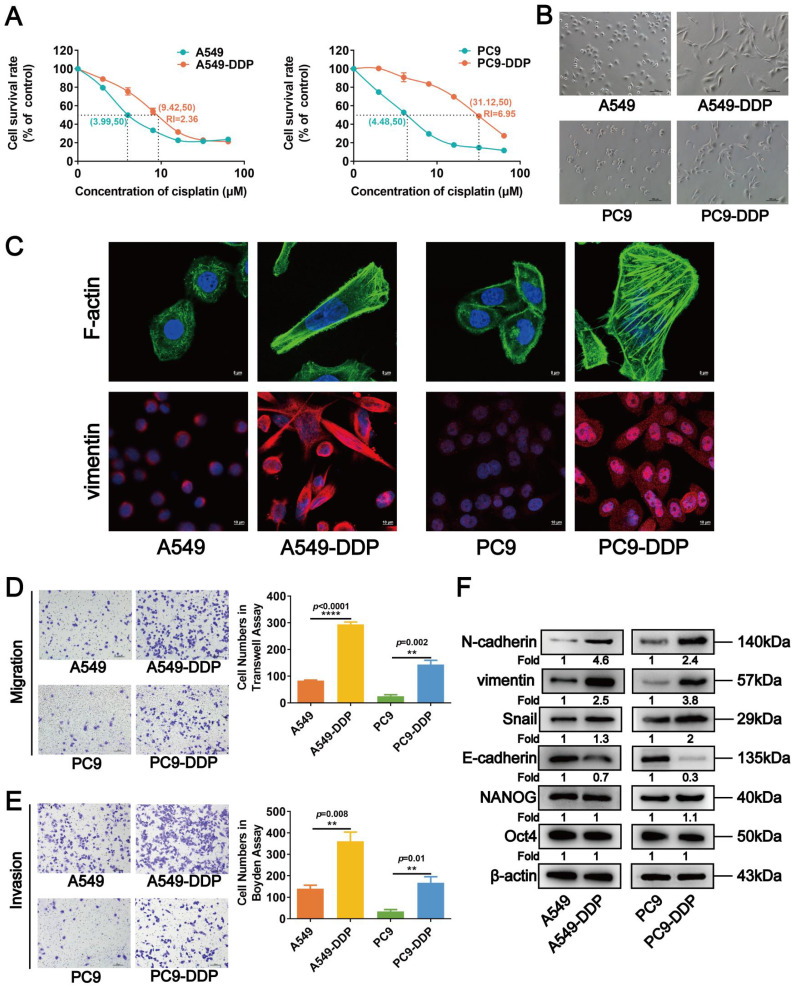
Cisplatin-resistant A549-DDP and PC9-DDP cells display EMT-like morphology and properties. (A) CCK8 assay to measure the half maximal inhibitory concentration (IC_50_) values of A549-DDP and PC9-DDP cell lines and their parental A549 and PC9 cells. RI, resistance index. (B) Morphology of the cisplatin-resistant LUAD cells and the corresponding wild type cell lines under microscope. Scar bars indicate 100μm. (C) F-actin and vimentin in cisplatin-resistant and the parental LUAD cells were visualized by fluorescence microscopy. Images were captured using a 63× objective lens. Scar bars indicate 5μm or 10μm. (D, and E) Transwell chamber (D) and Boyden chamber (E) assays were performed to evaluate the migration and invasion capacity of A549-DDP and PC9-DDP cells. The numbers of migrated and invaded cells were quantitated. Scar bars indicate 100μm. Error bars, mean±SEM. **p <0.01, ****p<0.0001. (F) The expression levels of EMT and stem-related proteins in cisplatin-resistant LUAD cells were detected by Western blot analyses. β-actin was used as a loading control.

**Figure 2:**
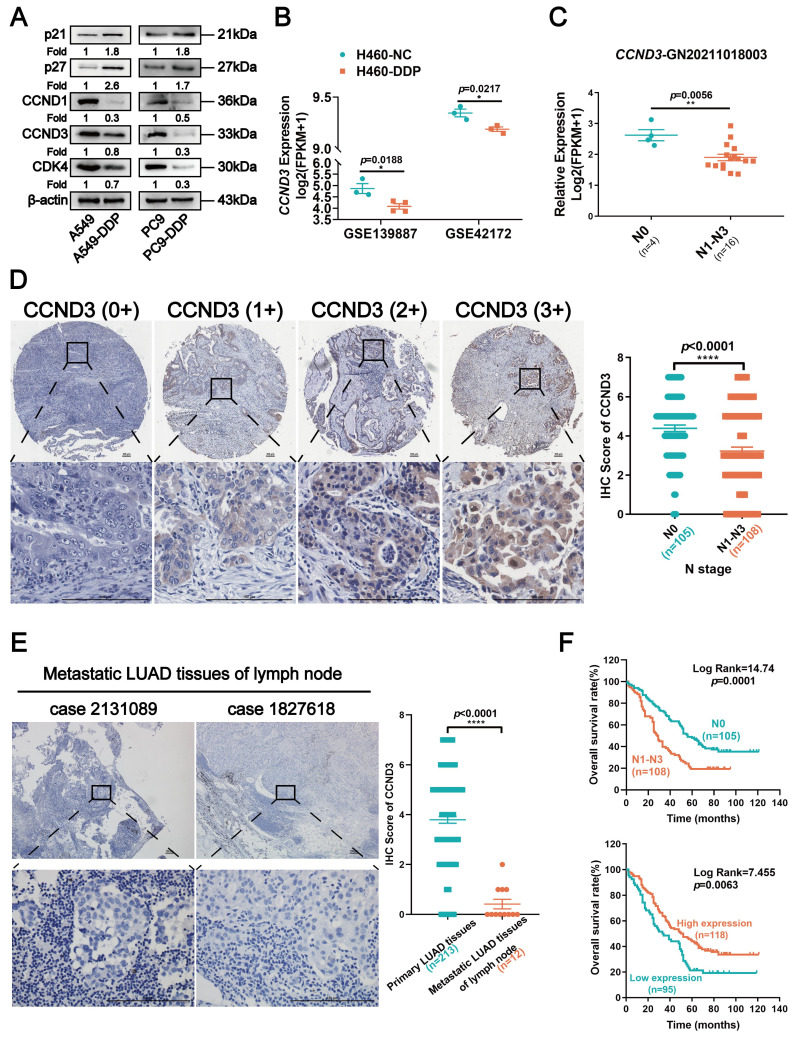
Downregulated CCND3 in cisplatin-resistant LUAD is associated with metastasis. (A) Western blot analysis of cell cycle-related proteins p21, p27, CCND1, CCND3, and CDK4 in cisplatin-resistant and parental LUAD cells. β-actin was used as a loading control. (B) Distribution of Fragments Per Kilobases per Million reads (FPKM) values of *CCND3* gene in H460-NC and H460-DDP cells based on the GEO datasets GSE139887 and GSE42172, respectively. Error bars, mean±SEM. *p<0.05. (C) Dot plot showing the expression of *CCND3* with the log2(FPKM+1) in a high-throughput RNA sequencing dataset GN20211018003 derived from tumors of 20 LUAD patients. N0: no lymph node metastasis; N1-N3: lymph node metastasis). Error bars, mean±SEM. **p<0.01. (D) Immunohistochemistry (IHC) staining of CCND3 protein in primary LUAD tissues. The first row: magnification, 4×; the second row: magnification, 40×. Scar bars indicate 100μm. Dot plot showing the IHC scores of CCND3 in LUAD patients at the N0 stage or the N1-N3 stages. Error bars, mean±SEM. ****p<0.0001. (E) IHC staining of CCND3 protein in metastatic LUAD of lymph node specimens. The first row: magnification, 4×; the second row: magnification, 40×. Scar bars indicate 100μm. Dot plot showing the IHC scores of CCND3 in the metastatic LUAD tissues of lymph node compared to that in the primary LUAD tissues. Error bars, mean±SEM. ****p<0.0001. (F) Kaplan-Meier survival curve showing the overall survival of 213 LUAD patients based on N stage (Top). The overall survival rates of LUAD patients with low or high expression level of CCND3 was estimated using the Kaplan-Meier analysis (Below).

The acquired DDP-resistant LUAD cells A549-DDP and PC9-DDP were established by gradually treating A549 and PC9 cells with DDP (initial concentrations of 1.33 and 1.67 μM, respectively). The IC_50_ values of the A549-DDP and PC9-DDP cells increased to 9.42 and 31.12 µM, respectively, after 8 months of incubation with increasing DDP concentrations, whereas their parental A549 and PC9 cells had IC_50_ values of 3.99 and 4.48 µM, respectively (Fig. 1A). We observed that the A549-DDP and PC9-DDP cells were more fusiform and formed more pseudopodia compared with their parental cells, showing the mesenchymal characteristics of DDP-resistant LUAD cells (Fig. 1B). The actin cytoskeletal organization of DDP-resistant cells was assessed by staining F-actin with FITC-phalloidin to further evaluate their EMT-like phenotype. As shown in Figure 1C, numerous pseudopodia with broadly parallel bundles of stress fibers appeared in A549-DDP and PC9-DDP cells. Furthermore, vimentin, a key intermediate filament protein and a mesenchymal marker, markedly accumulated in the cytoplasm of DDP-resistant LUAD cells (Fig. 1C). Transwell and Boyden chamber assays subsequently revealed significant increases in the migrative and invasive abilities of A549-DDP and PC9-DDP cells compared with the parental cell lines (Fig. 1D and 1E). Western blot analysis also revealed that the expression levels of several EMT-related proteins (N-cadherin, vimentin, and Snail) were upregulated, whereas that of E-cadherin was downregulated in A549-DDP and PC9-DDP cells (Fig. 1F), further validating a change in EMT in DDP-resistant LUAD cells. The cell proliferation was examined to further clarify the effect of DDP resistance on LUAD cells. The proliferation rate of A549-DDP and PC9-DDP cells significantly decreased compared with those of their parental counterparts (Fig. S1A). The results of EdU and flow cytometry assays revealed that the numbers of S-phase cells in the DDP-resistant cell lines were significantly lower than those in A549 and PC9 cells (Fig. S1B), with retardation of the cell cycle in the G2/M phase (Fig. S1C). Western blot analyses showed that the levels of cell cycle-related proteins p21 and p27 were upregulated in DDP-resistant cells, whereas those of CCND1, CCND3, and CDK4 were downregulated (Fig. 2A). The levels of tumor stem-related proteins, including NANOG and Oct4, showed no significant changes (Fig. 1F). Collectively, these results suggested that DDP-resistant LUAD cells acquired an EMT-like phenotype along with its associated properties, but their proliferation was restrained due to cell cycle arrest.

### CCND3 was downregulated in DDP-resistant LUAD and associated with its metastasis

The bioinformatic analysis was first performed to evaluate the alterations in the expression of *CCND3* during DDP treatment to explore the roles of CCND3 in DDP-resistant LUAD cells. As shown in Figure 2B, *CCND3* mRNA expression decreased in DDP-resistant H460-DDP cells (based on the GEO datasets GSE139887 and GSE42172), which was consistent with the aforementioned data showing reduced protein level of CCND3 in the DDP-resistant A549-DDP and PC9-DDP cell lines.

Another analysis using an RNA sequencing dataset (GN20211018003) derived from 20 LUAD specimens revealed that *CCND3* expression was higher in the 4 LUAD cases without lymph node metastasis but lower in the 16 LUAD cases with lymphatic spread (Fig. 2C). Furthermore, IHC was performed in 213 primary LUAD specimens and 12 metastatic LUAD of lymph node specimens to determine CCND3 protein expression. As shown in Figure 2D, CCND3 was predominantly expressed in the cytoplasm of LUAD cells. The expression level of CCND3 in patients with LUAD in the N0 stage was substantially higher compared with the expression levels corresponding to the N1 to N3 stages. Specifically, 65 (60.2%) of 108 LUAD cases with lymph node involvement exhibited CCND3 downregulation, whereas only 30 (28.6%) of 105 cases without lymphatic metastasis exhibited low CCND3 expression, indicating a significant correlation between the reduced expression of CCND3 and the presence of lymphatic metastases in patients with LUAD (*χ*^2^ = 21.535, *p* < 0.0001; Table S5). Significant differences were also observed in the correlation between CCND3 expression and clinical stage (*χ*^2^ = 29.675, *p* < 0.0001) as well as the M stage (*χ*^2^ = 4.519, *p* = 0.034) (Table S5). Moreover, CCND3 expression was evaluated in 12 cases of metastatic LUAD of lymph nodes compared with 213 cases involving primary LUAD tissues. The results showed low CCND3 expression in all 12 specimens of metastatic LUAD in the lymph nodes. In contrast, only 95 (44.6%) of 213 cases of primary LUAD exhibited low CCND3 expression (Fig. 2E and Table S6), further indicating a crucial role of CCND3 downregulation in LUAD metastasis. It was notable that LUAD patients with lymphatic metastasis had poorer overall survival ( *p* = 0.0001; Fig. 2F). Furthermore, the Kaplan-Meier analysis revealed that reduced CCND3 expression in LUAD was significantly correlated with worse overall survival rates in patients (*p*= 0.0063; Fig. 2F). In conclusion, these results showed a downregulation of CCND3 in DDP-resistant LUAD cells. CCND3 expression was negatively associated with lymphatic metastasis in patients with LUAD and thus positively associated with overall survival.

### Knockdown of CCND3 promoted chemoresistance and metastatic potential but reduced the proliferation of LUAD cells

**Figure 3:**
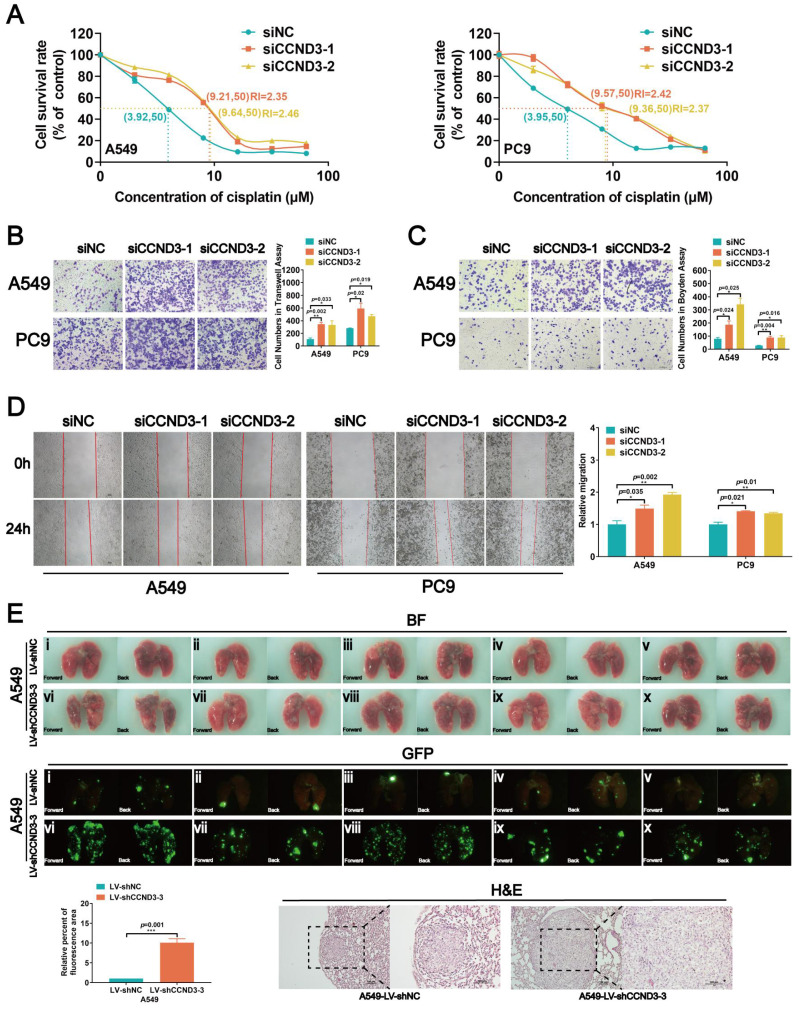
CCND3 depletion promotes cisplatin resistance and metastasis of LUAD cells. (A) The IC_50_ value of cisplatin was measured in CCND3-depleted A549 and PC9 cells. RI, resistance index. Error bars, mean±SEM. (B, C, and D) Transwell chamber assay, Boyden chamber assay and wound-healing assays were performed in CCND3-depleted A549 and PC9 cells and the corresponding control cells. Scar bars indicate 100μm. Error bars, mean±SEM. *p<0*.*05, **p<0*.*01. (E) An *in vivo* pulmonary metastasis model was generated to investigate the impact of CCND3 on LUAD metastasis (n = 5 per group). Bioluminescence images of the lungs were captured after tail vein injection of A549 cells. Error bars, mean±SEM. ***p<0.001. Lung metastases were confirmed by H&E staining. Scar bars indicate 100μm.

Either siRNAs targeting CCND3 or CCND3 plasmids were respectively transfected into parental and DDP-resistant LUAD cells to transiently knockdown or overexpress CCND3 so as to investigate the specific roles of CCND3 in LUAD (Figs. S2A and S3A). The CCK8 assay for the IC_50_ values revealed that CCND3 downregulation restrained the chemosensitivity of LUAD cells and caused a significant increase in the IC_50_ values (Fig. 3A). It was consistently found in DDP-resistant LUAD cells that CCND3 overexpression reduced the IC_50_ value to 32.56% in A549-DDP cells and to 63.66% in PC9-DDP cells (Fig. S3B). The results of the Transwell chamber assay revealed that the numbers of migrated cells increased in CCND3-silenced A549 and PC9 cells but decreased in CCND3-overexpressing A549-DDP and PC9-DDP cell lines (Figs. 3B and S3D). The wound healing assay further confirmed that CCND3 depletion accelerated the migration and recolonization of LUAD cells entering the wound area compared with controls, whereas this trend was reversed in DDP-resistant LUAD cells with CCND3 overexpression (Figs. 3D and S3E). The results of the Boyden chamber assay showed that CCND3 knockdown significantly enhanced the invasive capacity of A549 and PC9 cells (Fig. 3C), whereas CCND3 overexpression impeded the invasion of A549-DDP and PC9-DDP cells (Fig. S3F). An *in vivo* experiment was further performed to validate the effect of CCND3 on LUAD metastasis. Lentivirus-mediated shRNAs for CCND3 carrying green fluorescent protein (GFP) were introduced to establish LUAD cell lines with stably silenced CCND3 (Fig. S2B and S2C). The lungs of nude mice were removed and photographed under fluorescence microscopy 6 weeks after the intravenous injection of A549-LV-shCCND3 cells. The results revealed that CCND3-depleted LUAD cells were widely dispersed and formed more metastases in the lung compared with the control group (Fig. 3E). These data suggested that CCND3 depletion augmented the migration, invasion, and metastasis of LUAD cells. Previous studies have suggested that CCND3 accelerates cell cycle progression across various tumors 21-23. Therefore, a series of proliferation assays were conducted to evaluate the role of CCND3 in LUAD cell growth. The CCK8 assay results showed that LUAD cell growth was impaired after CCND3 knockdown (Fig. S2D). In contrast, the DDP-resistant cell lines proliferated more rapidly after CCND3 overexpression (Fig. S3G). An EdU experiment showed that the percentage of S-phase LUAD cells significantly decreased after CCND3 depletion (Fig. S2E), whereas the percentage of S-phase DDP-resistant cells increased after CCND3 overexpression (Fig. S3H). Flow cytometry detected a reduction in the proportion of S-phase cells and proliferative index alongside an increase in the proportion of G0/G1 phase cells following the inhibition of CCND3 expression (Fig. S2F), thereby indicating that CCND3 inhibition blocked the G1-to-S cell cycle transition. Collectively, the results revealed a dual role for CCND3 depletion, whereby it promoted migration, invasion, metastasis, and chemoresistance to DDP while concurrently restraining cell proliferation in LUAD.

### LUAD cells with CCND3 depletion displayed an EMT-like phenotype

**Figure 4:**
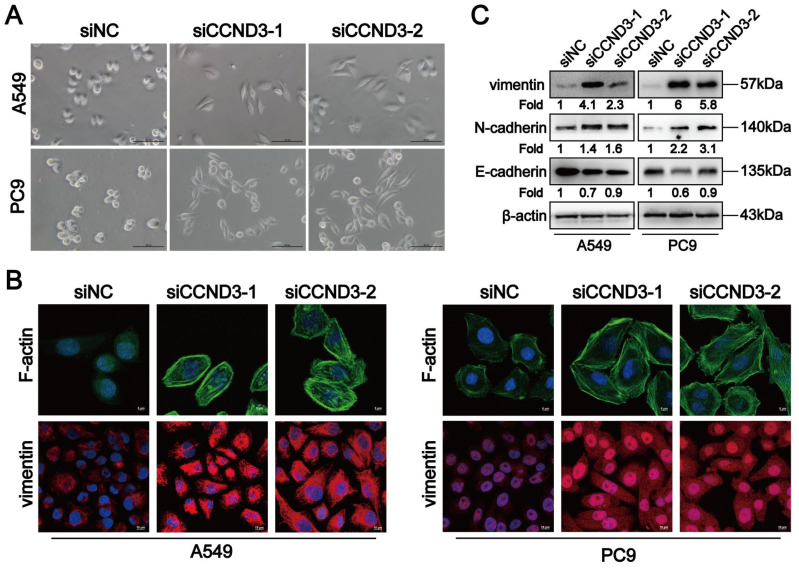
CCND3 depleted LUAD cells manifest an EMT-like phenotype. (A) The morphology of A549 and PC9 cells were photographed after CCND3 knockdown. Scar bars indicate 100μm. (B) The distribution of F-actin and vimentin in CCND3-silenced LUAD cells was captured under immunofluorescence microscopy. Images were captured using with a 63× objective lens. Scar bars indicate 5 or 10μm. (C) Changes of vimentin, N-cadherin and E-cadherin protein levels in A549 and PC9 cell lines were detected by Western blot after the transfection of CCND3 siRNAs. β-actin was used as a loading control.

While exploring the role of CCND3 in LUAD, we observed that some of the A549 and PC9 cells were more elongated after knocking down CCND3 (Fig. 4A). On the contrary, the morphology of DDP-resistant A549-DDP and PC9-DDP cells changed from spindle-shaped to more oval-shaped following CCND3 overexpression (Fig. S3C). Furthermore, as shown in Figure 4B, the stress fibers in CCND3-silenced A549 and PC9 cells appeared as lamellipodia, which were densely stained and exhibited a well-organized structure. In addition, CCND3 depletion increased the expression of vimentin and N-cadherin in A549 and PC9 cells, whereas it inhibited the protein expression of E-cadherin (Fig. 4B and 4C). Conversely, CCND3 overexpression attenuated the expression of vimentin and N-cadherin while simultaneously increasing the expression of E-cadherin in DDP-resistant A549-DDP and PC9-DDP cell lines (Fig. S3I). Overall, these data indicated that CCND3 depletion facilitated EMT of LUAD cells.

### CCND3 accelerated the degradation of vimentin by recruiting PARK2

**Figure 5:**
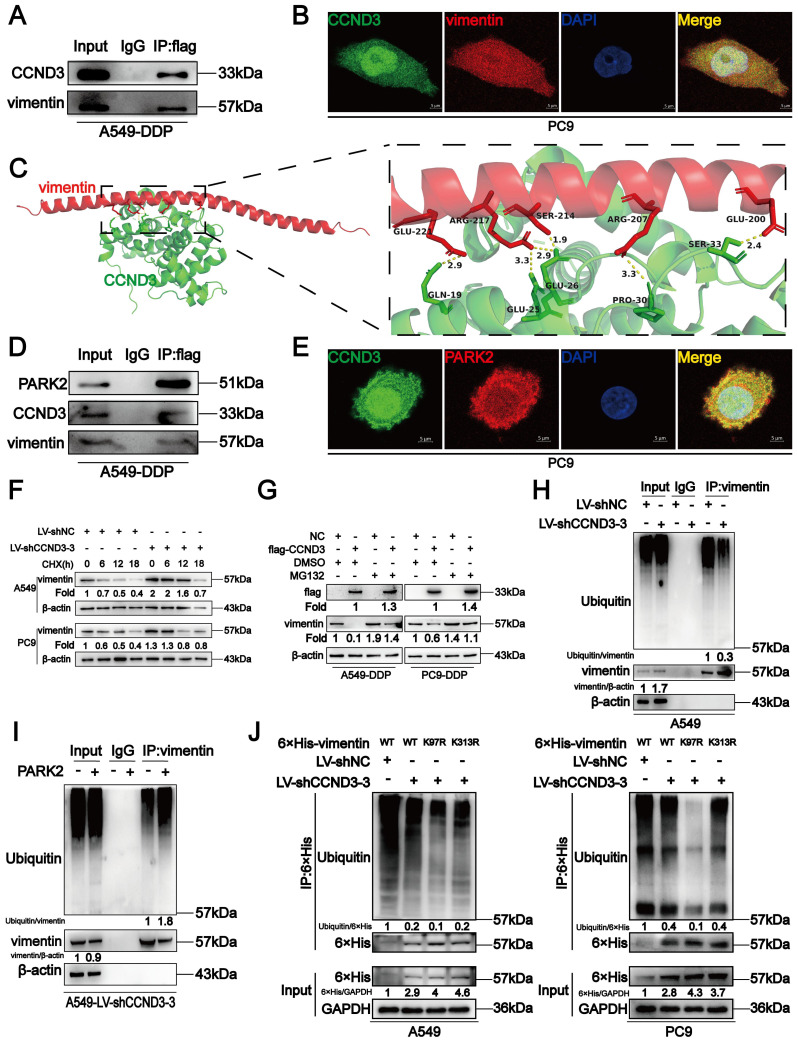
CCND3 recruits PARK2 to degrade vimentin. (A) The binding of CCND3 and vimentin was verified by co-immunoprecipitation assay in A549-DDP cells. Input was served as a positive control and irrelevant IgG was the negative control. (B) Co-localization of CCND3 and vimentin was detected by the analysis of dual immunofluorescence staining (CCND3, green; vimentin, red) in PC9 cells. Scar bars indicate 5μm. (C) The predicted binding mode of vimentin [Protein Data Bank (PDB) entry 4YPC chain A; red] to the domain of CCND3 (PDB entry 7SJ3 chain B; green). The hydrogen bond between the two molecules was drawn in yellow dotted line. (D) Co-immunoprecipitation analysis indicated the interaction between CCND3 and PARK2 in A549-DDP cells. (E) Immunofluorescence costaining of CCND3 (green) and PARK2 (red) in PC9 cells. Scar bars indicate 5μm. (F) Western blot analyses evaluated the impact of CCND3 depletion on vimentin stability in A549 and PC9 cells incubated with cycloheximide (CHX) at the indicated time points. β-actin was used as a loading control. (G) Western blot analyses demonstrated the effect of CCND3 overexpression on vimentin stability in A549-DDP and PC9-DDP cells incubated with DMSO or MG132. β-actin was used as a loading control. (H, I) Co-immunoprecipitation analyses detected the ubiquitination of vimentin after CCND3 knockdown (H) or PARK2 overexpression (I). β-actin was used as a loading control. (J) Co-immunoprecipitation to identify the ubiquitination site on vimentin. A549 and PC9 cells were transfected with wild-type or mutant 6×His-vimentin (K97R or K313R). The 6×His-tagged proteins in the cell lysate were affinity-purified and probed with Ub antibody and 6×His antibody in Western blot analyses. GAPDH was used as a loading control.

We screened proteins interacting with CCND3 to investigate the underlying molecular mechanism by which CCND3 impeded EMT and metastasis in DDP-resistant LUAD. Co-IP was performed in A549-DDP cells to detect which proteins interacted with CCND3. We visually distinguished three differential bands at approximately 35-40, 55-70, and 100 kDa between the IP and IgG groups using polyacrylamide gels with silver staining. Of these, the band of approximately 55-70 kDa in the IP group showed the most distinct difference compared with its corresponding IgG group (Fig. S4A and S4B). The three differential bands were cut out from the gel with Coomassie brilliant blue staining and then sent for LC-MS/MS. Intriguingly, the canonical mesenchymal marker vimentin was identified as one of the probable candidates (Table S7). Co-IP was then conducted to verify whether an interaction existed between vimentin and CCND3. As displayed in Figure 5A, by transfecting flag-labeled GV141-CCND3 plasmids into A549-DDP cells (Fig. S4C), vimentin was pulled down after incubation with the anti-flag antibody, thereby confirming the binding between CCND3 and vimentin. In addition, the CCND3 and vimentin proteins were co-located in the nucleus and cytoplasm of PC9 cells by a double immunofluorescence staining assay (Fig. 5B). Moreover, protein-protein docking was performed using the HDOCK server. The top 10 models were analyzed through PyMOL to select the configuration with the maximum number of hydrogen bonds between the two molecules. In the configuration of protein complexes in Figure 5C, vimentin (PDB entry 4YPC chain A; red) formed six hydrogen bonds with CCND3 (PDB entry 7SJ3 chain B; green). These results indicated that CCND3 interacted with vimentin in LUAD. The BioGRID database was applied to predict their interacting proteins to further elucidate the regulatory relationship between CCND3 and vimentin. The E3 ubiquitin ligase PARK2 was identified as a candidate interacting protein of both CCND3 and vimentin. Next, flag-labeled PARK2 plasmids were transfected into A549-DDP cells (Fig. S4D) while preparing for the Co-IP assay to verify the interaction of PARK2 with CCND3 and vimentin (Fig. 5D). The immunofluorescence results further validated that CCND3 and PARK2 were expressed in both the nucleus and cytoplasm of LUAD cells with clear co-localization (Fig. 5E). These data revealed the potential for interaction among the CCND3, vimentin, and PARK2 proteins. Based on the aforementioned results showing the negative regulation of the protein level of vimentin by CCND3 (Figs. 4C and S3I), we preliminarily speculated that CCND3 exerted its anti-metastasis function by recruiting PARK2 to ubiquitinate and degrade vimentin in LUAD. This was confirmed by subsequent cycloheximide (CHX) and MG132 intervention trials. As shown in Figure 5F, CCND3-silenced and control LUAD cells were subjected to protein synthesis inhibitor CHX treatment for 0, 6, 12, and 18 h to detect the protein levels of vimentin at each time point. The protein levels of vimentin stabilized and its half-life was dramatically elongated after CCND3 depletion, indicating that the degradation of vimentin was attenuated by CCND3 silencing. Moreover, as shown in Figure 5G, vimentin was downregulated after CCND3 overexpression in DDP-resistant LUAD cells. In contrast, the addition of the proteasome inhibitor MG132 significantly abrogated CCND3-mediated vimentin inhibition, implying that CCND3 led to vimentin degradation via modulating the proteasome pathway. We performed ubiquitination analyses to verify whether PARK2 degraded vimentin. The results revealed that the ubiquitination of vimentin was impaired following CCND3 depletion, whereas the addition of PARK2 restored the ubiquitination-mediated degradation of vimentin caused by CCND3 knockdown (Fig. 5H and 5I). Further, we predicted in three databases that lysine 97 (K97) and lysine 313 (K313) residues on vimentin were potential key sites where CCND3-mediated ubiquitination via PARK2 occurred. We compared the ubiquitination levels of vimentin in LUAD cells transfected with VIM-6×His constructs carrying mutations for K97R or K313R to identify the importance of the aforementioned sites. The VIM-6×His construct with the K97R mutation manifested a reduced level of ubiquitination, strongly suggesting a crucial role of the K97 residue in regulating vimentin ubiquitination (Fig. 5J). In conclusion, these findings suggested that the recruitment of PARK2 by CCND3 led to the degradation of vimentin. Moreover, K97 was identified as a vital site for vimentin ubiquitination and its subsequent degradation.

### CCND3 impeded the migration and invasion of LUAD cells by inhibiting vimentin

**Figure 6:**
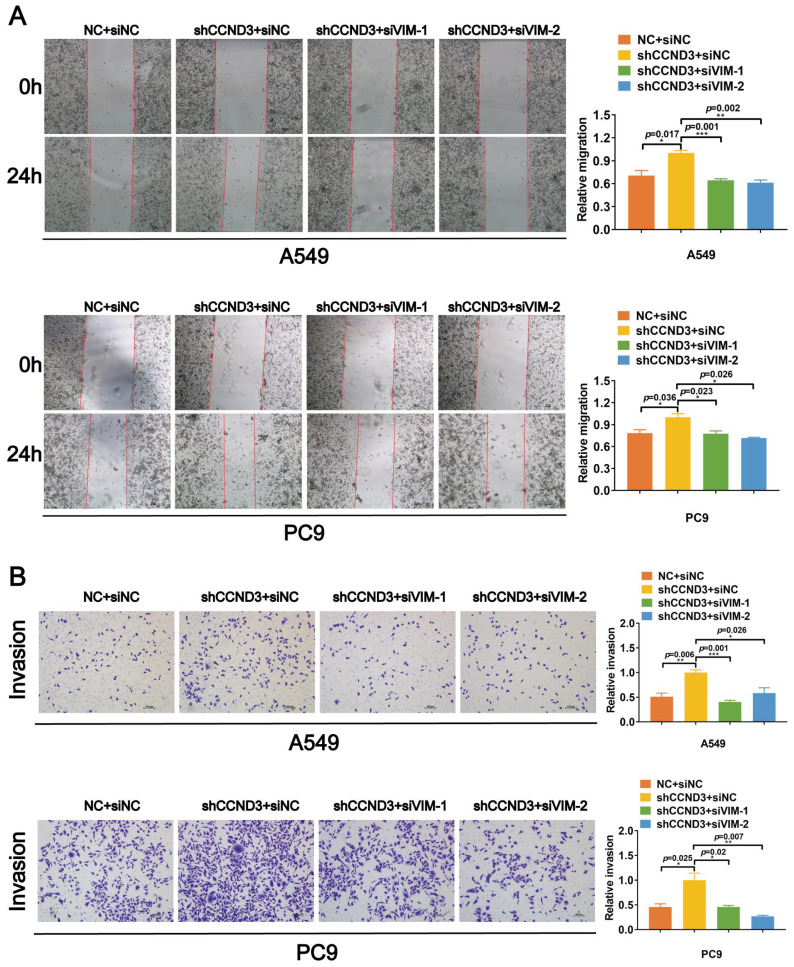
CCND3 attenuates the migration and invasion of LUAD cells by repressing vimentin. (A, and B) siRNAs targeting vimentin were transfected into CCND3 stably depleted A549 and PC9 cells to observe the migrative and invasive alteration by wound-healing assay (A) and Boyden chamber assay (B). Scar bars indicate 100μm. Error bars, mean±SEM. *p<0*.*05, **p<0.01, ***p<0.001.

We knocked down vimentin in LUAD cells with stably silenced CCND3 using two selected siRNAs with better interference effects targeting vimentin to confirm that CCND3 suppressed vimentin to restrain the migration and invasion of LUAD (Fig. S4E). Based on the wound healing and Boyden chamber assay results shown in Figure 6A and 6B, the migration and invasion of A549 and PC9 cells were significantly reinforced, whereas this effect was abrogated by vimentin depletion after the stable silencing of CCND3. Collectively, these results suggested that CCND3 attenuated the metastatic potential of LUAD by recruiting PARK2 to degrade vimentin.

### Reduction of CCND3 in DDP-resistant LUAD cells was attributable to negative regulation by PI3K/AKT/c-Jun

**Figure 7:**
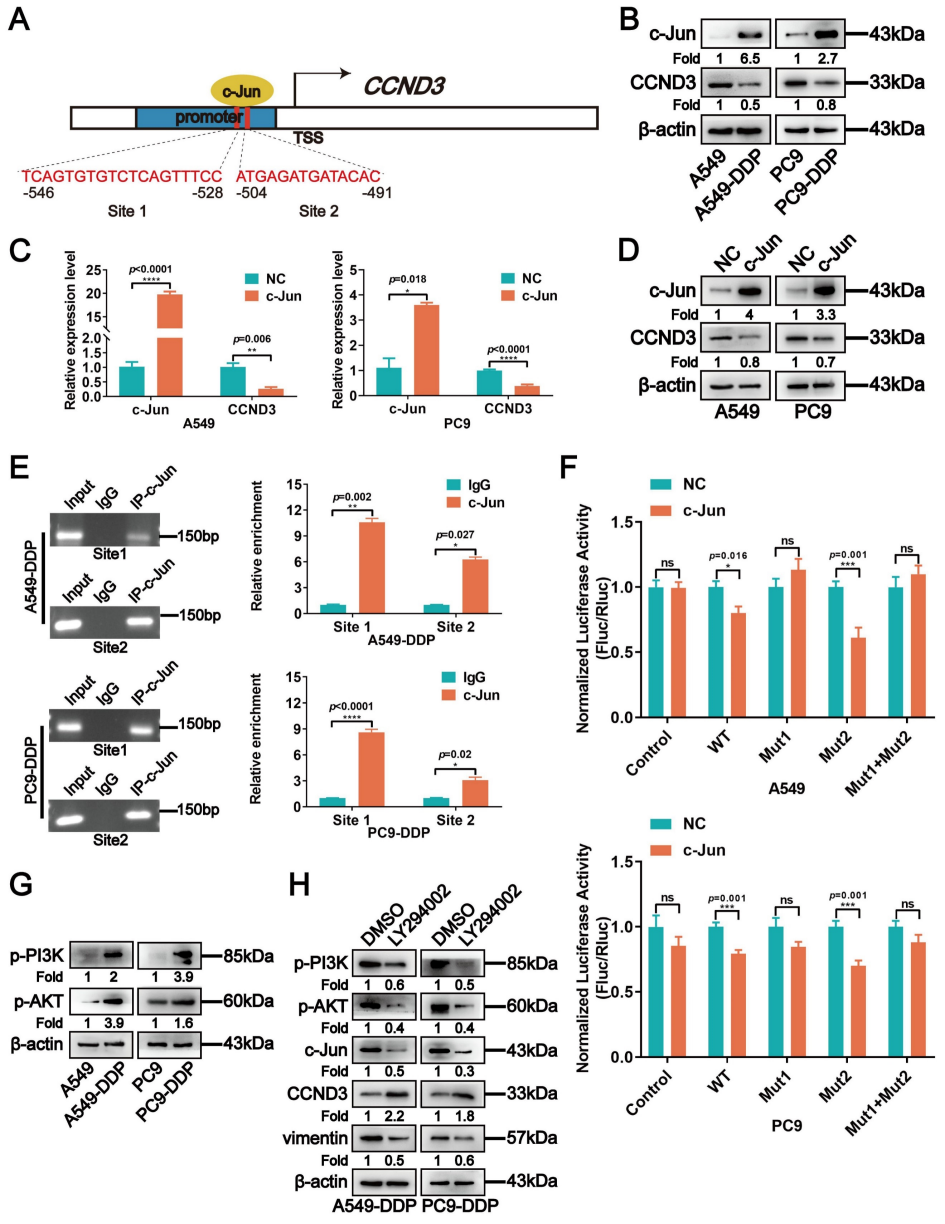
CCND3 depletion in cisplatin-resistant LUAD cells is attributed to the negative regulation by PI3K/AKT/c-Jun. (A) The predicted c-Jun binding sites to the promoter region of *CCND3*. (B) The expression levels of c-Jun and CCND3 were determined in cisplatin-resistant and parental LUAD cells by Western blot analyses. β-actin was used as a loading control. (C) mRNA expression of c-Jun and CCND3 in A549 and PC9 cells transfected with c-Jun plasmids was detected by qRT-PCR. Error bars, mean±SEM. *p<0.05, **p<0.01, ****p<0.0001. (D) The protein levels of c-Jun and CCND3 in A549 and PC9 cells transfected with c-Jun plasmids was evaluated by Western blot. β-actin was used as a loading control. (E) The chromatin immunoprecipitation assay to verify the binding sites of c-Jun to CCND3. Error bars, mean±SEM. *p<0.05, **p<0.01, ****p<0.0001. (F) The luciferase reporter assay was used to determine the binding of c-Jun to the promoter Site 1 of CCND3. Error bars, mean±SEM. ns represents no significance. *p<0.05, ***p<0.001. (G) The expression levels of p-PI3K and p-AKT proteins in cisplatin-resistant LUAD cells were detected by Western blot analyses. β-actin was used as a loading control. (H) Western blot detection of p-PI3K, p-AKT, c-Jun, CCND3, and vimentin expression in A549-DDP and PC9-DDP cell lines treated with PI3K inhibitor LY294002. β-actin was used as a loading control.

The LASAGNA and Jaspar algorithms were applied to screen for the upstream transcriptional regulation of *CCND3* to determine the underlying mechanism of CCND3 downregulation in DDP-resistant LUAD. We analyzed the 2000-kb sequence upstream of the transcription start site of *CCND3* to map its putative transcription factor binding profile. We found two potential binding sites of c-Jun located at -546 to -528 and -504 to -491, respectively, in the putative promoter region of CCND3 (Fig. 7A). Previous studies have suggested a major role of c-Jun in the mechanism of DDP resistance in various types of tumors 24. The present study found that the observed alteration in the c-Jun protein level was contrary to that of CCND3, as indicated by the upregulation in DDP-resistant A549-DDP and PC9-DDP cells compared with the parental cell lines (Fig. 7B). c-Jun plasmids were transduced into A549 and PC9 cells to detect the mRNA and protein levels of CCND3 to elucidate whether CCND3 was transcriptionally regulated by c-Jun. As shown in Figure 7C and 7D, both the mRNA and protein levels of CCND3 significantly decreased following c-Jun overexpression, indicating that c-Jun might act as an upstream inhibitor of CCND3 in LUAD. The ChIP assay was then performed to verify the binding of c-Jun to the promoter of CCND3. The results indicated that c-Jun bound to both sites 1 and 2 on the CCND3 promoter (Fig. 7E). Furthermore, co-transfection of c-Jun with either CCND3-WT or CCND3-Mut2 significantly reduced luciferase activity in A549 and PC9 cells, as verified by the dual-luciferase reporter assay. However, when c-Jun was co-transfected with either CCND3-Mut1 or the CCND3-Mut1+Mut2 plasmid, the luciferase activity was not significantly affected (Fig. 7F). These findings indicated that c-Jun transcriptionally inactivated CCND3 predominantly by binding to the promoter site 1 of CCND3. Recent studies reported that PI3K/AKT signaling participated in the progress of metastasis and DDP resistance in tumors 25-28. c-Jun was positively regulated by the PI3K/AKT pathway 18, 29-31. Therefore, we investigated the activation of the PI3K/AKT/c-Jun signaling pathway in DDP-resistant LUAD cells. The protein levels of p-PI3K and p-AKT were elevated in DDP-resistant cell lines compared with their parental cells (Fig. 7G). Moreover, the protein levels of p-PI3K, p-AKT, c-Jun, and vimentin decreased, whereas that of CCND3 increased in DDP-resistant LUAD cells following treatment with the PI3K inhibitor LY294002 (Fig. 7H). Overall, these findings suggested that DDP negatively regulated the transcription of CCND3 by activating the PI3K-AKT-c-Jun axis, thereby downregulating the expression of CCND3 in LUAD.

## Discussion

DDP is the most commonly used chemotherapeutic agent in patients with LUAD. However, it has been clinically observed that many patients with LUAD develop drug resistance after undergoing a period of DDP treatment, leading to treatment failure and a high incidence of metastasis. As a result, chemotherapeutic resistance and metastasis make therapy challenging for patients with LUAD. Exploring the possible mechanisms related to DDP resistance and metastasis in LUAD would, therefore, be of great significance for optimizing the treatment regimen.

Chemoresistance is classified into intrinsic and acquired resistance. However, studies on cases of acquired DDP resistance have been relatively few. Existing studies on tumors have reported that acquired DDP resistance is accompanied by alterations in EMT 32, 33. Our results were consistent with previous studies showing that DDP-resistant LUAD cells exhibited the morphological characteristic of the EMT phenotype, with cytoskeleton remodeling in conjunction with enhanced migrative and invasive potential compared with the corresponding parental cells. In contrast, the proliferation of DDP-resistant LUAD cells was markedly restrained, which was mainly attributed to the inhibition of G2/M phase transition. The subsequent mechanistic analysis demonstrated that the EMT signal was significantly reinforced in DDP-resistant LUAD cells, whereas the cell cycle signaling was notably weakened, including the reduced expression of CCND3. These findings were similar to the previously reported changes in tumors following DDP resistance 34. However, some tumor stemness markers did not exhibit significant alterations in their expression. These data suggested that acquired DDP-resistant LUAD cells predominantly had cell cycle signal blocking and EMT signal activation.

In previous studies, increased CCND1 and CCND2 expression was found to promote cell proliferation, migration, invasion, and intrinsic chemoresistance 35-41. As a member of the D-type cyclin family regulating the transition of the cell cycle, CCND3 has been reported to promote cell growth in various types of tumors 14, 21, 42-46; however, its roles in tumor chemotherapeutic resistance and tumor metastasis remain unexplored. Thus, we investigated the role of CCND3 in acquired chemoresistance and LUAD metastasis. In line with the previous studies, we first confirmed the role of CCND3 in promoting tumor growth. Unexpectedly, we observed that CCND3-depleted LUAD cells exhibited a mesenchymal-like phenotype, as well as a shift in cytoskeletal dynamics. Subsequent data revealed that the reduction of CCND3 promoted cell migration, invasion, metastasis, and chemoresistance to DDP via increasing vimentin and N-cadherin levels in LUAD. These data elucidated that CCND3 promoted cell growth but significantly suppressed metastatic potential via inactivating the EMT signal and triggering cytoskeleton remodeling in acquired DDP-chemoresistant LUAD, thus suggesting a new role of CCND3 as a metastatic suppressor in tumors.

We screened CCND3-interactive proteins using Co-IP coupled with mass spectrometry in A549-DDP cells to explore the molecular mechanism of CCND3 in impeding LUAD metastasis. Vimentin, which is a canonical mesenchymal marker involved in DDP-induced tumor chemotherapy resistance 47, 48, was verified as an interacting protein of CCND3 by Co-IP and immunofluorescence assays. Previous experiments found that CCND3 negatively modulated the protein expression of vimentin in LUAD cells. Yet, the detailed molecular basis by which CCND3 decreased the protein expression of vimentin remained unclear.

Based on the BioGRID database, we found that PARK2, a suppressive E3 ubiquitin ligase in tumors 49, 50, was a potential interactive protein of both CCND3 and vimentin. Therefore, we speculated that CCND3 might recruit PARK2 to ubiquitinate and degrade vimentin in LUAD. In subsequent studies, we confirmed their interactive combinations and co-localization in the cytoplasm and nucleus, thereby validating that CCND3 mediated the ubiquitination and degradation of vimentin protein via recruiting PARK2. These data explained why downregulated CCND3 promoted LUAD metastasis and chemotherapeutic resistance.

The present study revealed that CCND3 was downregulated in acquired DDP-resistant LUAD cells. However, the molecular mechanism of CCND3 downregulation remained to be elucidated. The key oncogenic PI3K/AKT signal 24, 51 and its downstream oncogenic transcription factor c-Jun 24, 52-54 are activated in acquired DDP-resistant tumors, including LUAD. Further, c-Jun was predicted to bind to the promoter of *CCND3*. We then hypothesized that PI3K/AKT activation induced c-Jun expression, which might subsequently bind to the *CCND3* promoter and suppress its expression in acquired DDP-resistant LUAD cell lines. Consistent with this speculation, we observed that c-Jun bound to *CCND3* at the -546 to -528 position in the putative promoter region and transcriptionally downregulated CCND3 expression. Further, the PI3K inhibitor LY294002 successfully reduced the activity of the PI3K/Akt/c-Jun signaling pathway and restored the expression of CCND3 in DDP-resistant LUAD cell lines.

We observed a reduced level of *CCND3* in LUAD samples in the N1-N3 stage compared with the N0 stage in clinical specimens based on high-throughput mRNA expression data. We further validated the aforementioned findings by analyzing CCND3 expression via IHC in LUAD tissue samples. Our results consistently demonstrated a significant negative relationship between CCND3 expression levels and the N stage, which is a clinical parameter for lymphatic metastasis. The IHC assay in metastatic lymphatic LUAD specimens further validated that CCND3 downregulation was a significant event in the lymphatic spread of LUAD. The survival analysis showed that LUAD patients with higher CCND3 expression gained significantly better survival outcomes compared with those with lower CCND3 expression.

In summary, this study revealed a novel role for CCND3 downregulation in promoting the metastatic potential of LUAD with acquired DDP chemoresistance. The molecular basis for this phenomenon is that downregulated CCND3 inhibits the recruitment of PARK2 to ubiquitinate and degrade vimentin, thus inducing EMT, further metastasis, and chemotherapeutic resistance to DDP in LUAD. In addition, the activation of the PI3K/AKT/c-Jun signaling pathway is a notable event involved in the downregulation of CCND3 in acquired DDP-chemoresistant LUAD. Furthermore, CCND3 downregulation in patients with LUAD is associated with lymphatic metastasis and poor prognosis, highlighting its important contribution to LUAD.

## Supplementary Material

Supplementary figures.

## Abbreviations

CCND3: Cyclin D3
DDP: cisplatin
LUAD: lung adenocarcinoma
EMT: epithelial-mesenchymal transition
IC_50_: half maximal inhibitory concentration
CCK-8: Cell Counting Kit-8
siRNAs: small interfering RNAs
VIM: vimentin
shRNA: short hairpin RNA
Co-IP: Co-immunoprecipitation
LC-MS/MS: liquid chromatography-tandem mass spectrometry
ChIP: Chromatin immunoprecipitation
IHC: immunohistochemistry
GFP: green fluorescent protein
CHX: cycloheximide
K97: lysine 97
K313: lysine 313
