# Supplementary material for: Downregulated CCND3 Is a Key Event Driving Lung Adenocarcinoma Metastasis during Acquired Cisplatin Resistance
Source: Int J Biol Sci. 2025 Jan 1;21(2):708–24. doi: 10.7150/ijbs.100921 (PMC11705650; doi:10.7150/ijbs.100921)
Supplement: Supplementary file 1 — Supplementary figures. [file ijbsv21p0708s1.pdf]

Fig. S1

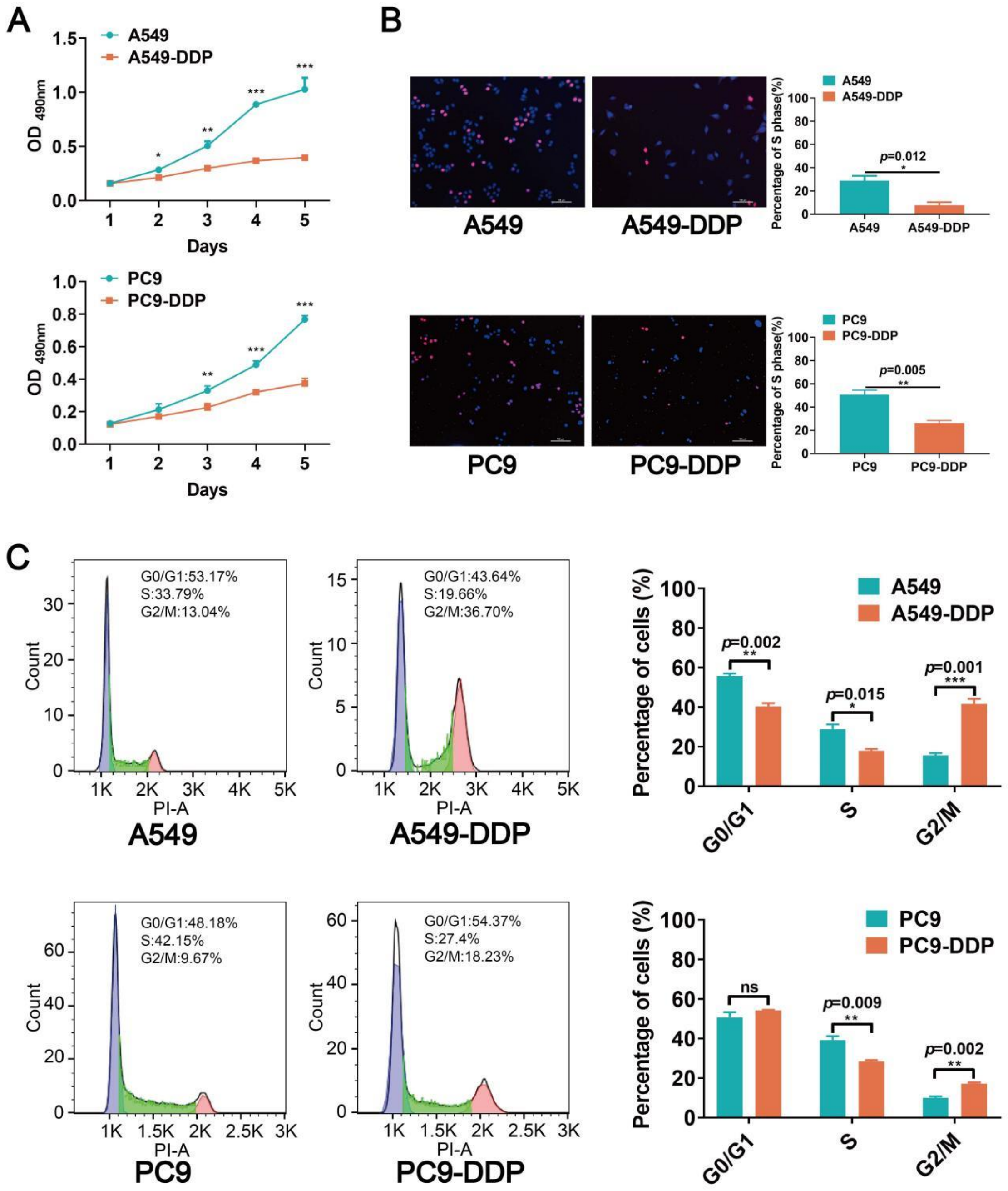

Fig. S1 The proliferative capacity of cisplatin-resistant LUAD cells. (A) The proliferation of cisplatin-resistant and parental cells was measured using Cell Counting Kit-8 (CCK8) assay. Error bars, mean±SEM. **p*<0.05, ***p*<0.01, ****p*<0.001. (B) EdU staining (EdU, red; DAPI, blue) of cisplatin-resistant and parental LUAD cells. Scar bars indicate 100μm. Error bars, mean±SEM. **p*<0.05, ***p*<0.01. (C) Flow cytometry was used to detect the cell cycle of A549-DDP, PC9-DDP and their corresponding parental cell lines. Error bars, mean±SEM. ns represents no significance. **p*<0.05, ***p*<0.01, ****p*<0.001.

Fig. S2

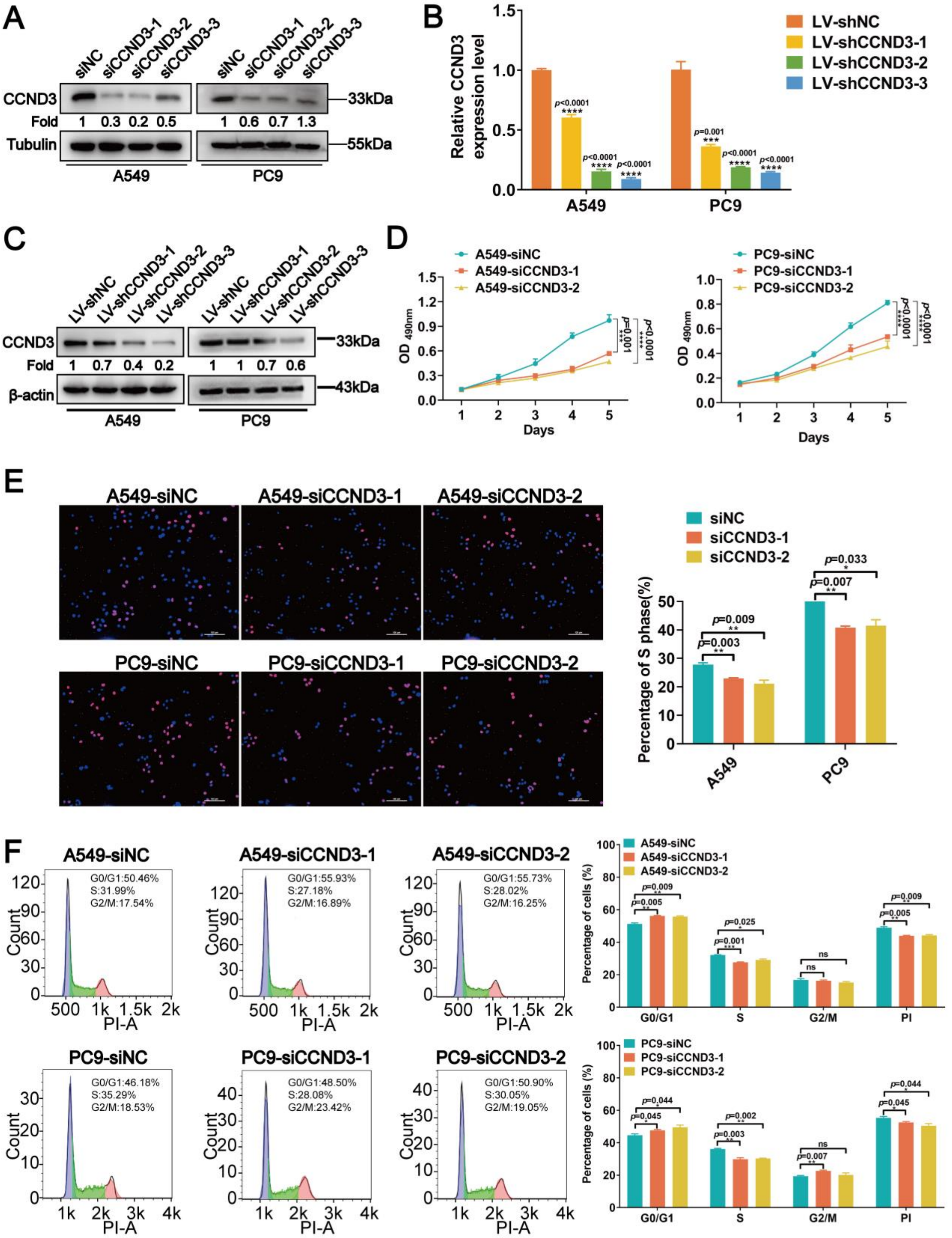

Fig. S2 The identification of CCND3 knockdown and the proliferation of CCND3-depleted LUAD cells. (A) The knockdown efficiency of three siRNAs targeting CCND3 in A549 and PC9 cells was evaluated by Western blot analysis. Tubulin was used as a loading control. (B, and C) The knockdown efficiency of lentivirus-mediated short hairpin RNAs targeting CCND3 in A549 and PC9 cells was evaluated by qRT-PCR (B) and Western blot analyses (C, β-actin was used as a loading control.). Error bars, mean±SEM. ***p<0.001, ****p<0.0001. (D, E, and F) The proliferation of CCND3-depleted A549 and PC9 cells was measured by CCK8

assay (D), EdU assay (E) and Cell cycle analysis (F). Scar bars indicate 100μm. Error bars, mean±SEM. ns represents no significance. *p<0.05, **p<0.01, ***p<0.001, ****p<0.0001.

Fig. S3

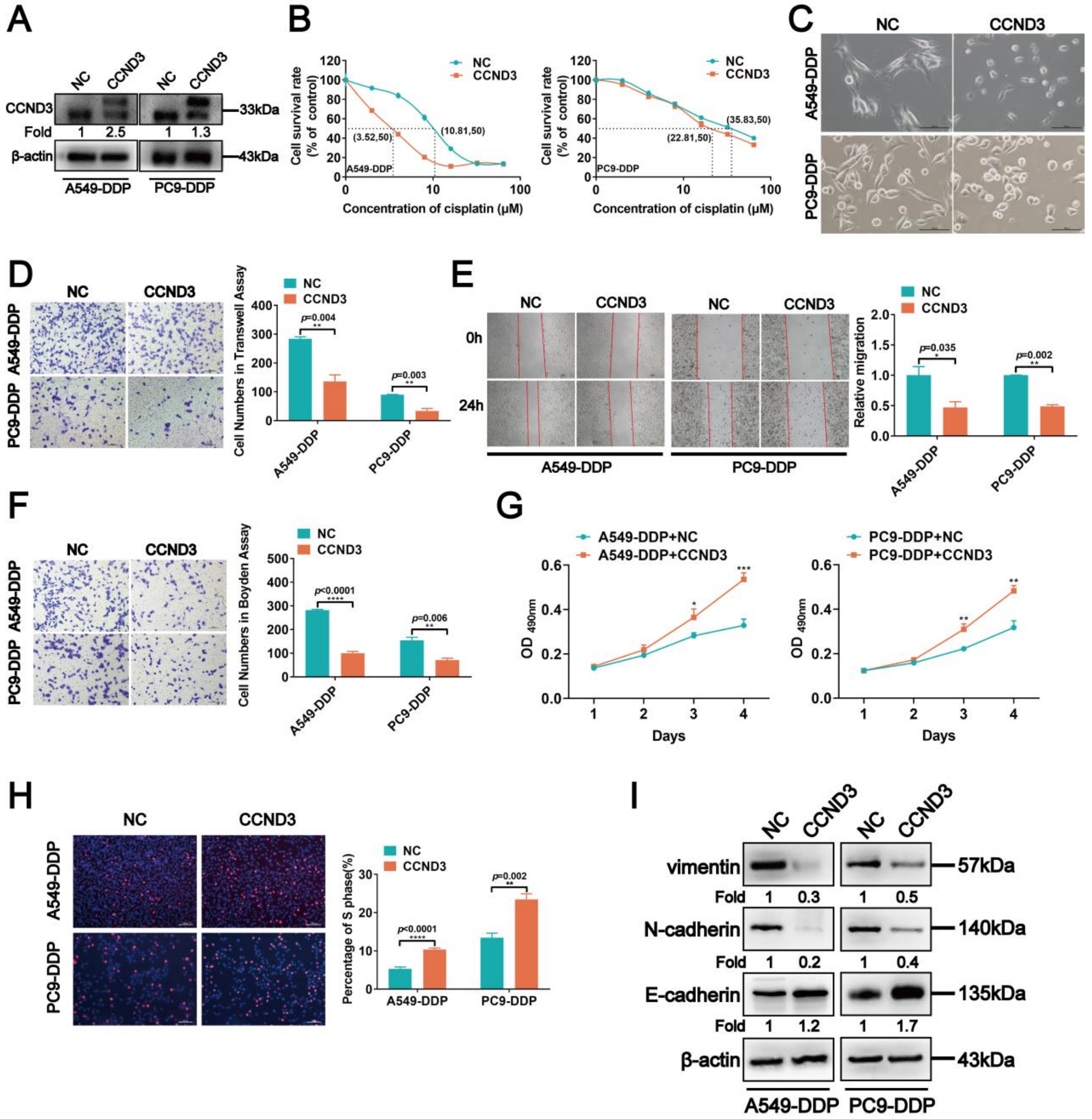

Fig. S3 The phenotypes of CCND3-overexpressed A549-DDP and PC9-DDP cells. (A) The efficiency of CCND3 overexpression in cisplatin-resistant LUAD cells was evaluated by Western blot analysis. β-actin was used as a loading control. (B) The IC_{50} value of cisplatin was measured in CCND3-overexpressed A549-DDP and PC9-DDP cells. Error bars, mean±SEM. (C) The morphology of cisplatin-resistant LUAD cells after CCND3 overexpression. Scar bars indicate 100μm. (D, E, and F) Transwell chamber assay (D), wound-healing assay (E) and Boyden chamber assay (F) in CCND3-overexpressed A549-DDP and PC9-DDP cells. Scar bars indicate 100μm. Error bars, mean±SEM. *p<0.05, **p<0.01, ****p<0.0001. (G, and H) CCK8 (G) and EdU assay (H) in CCND3-overexpressed A549-DDP and PC9-DDP cells. Scar

bars indicate 100μm. Error bars, mean±SEM. *p<0.05, **p<0.01, ***p<0.001, ****p<0.0001. (I) Protein levels of vimentin, N-cadherin, and E-cadherin in CCND3-overexpressed A549-DDP and PC9-DDP cells were detected by Western blot analysis. β-actin was used as a loading control.

Fig. S4

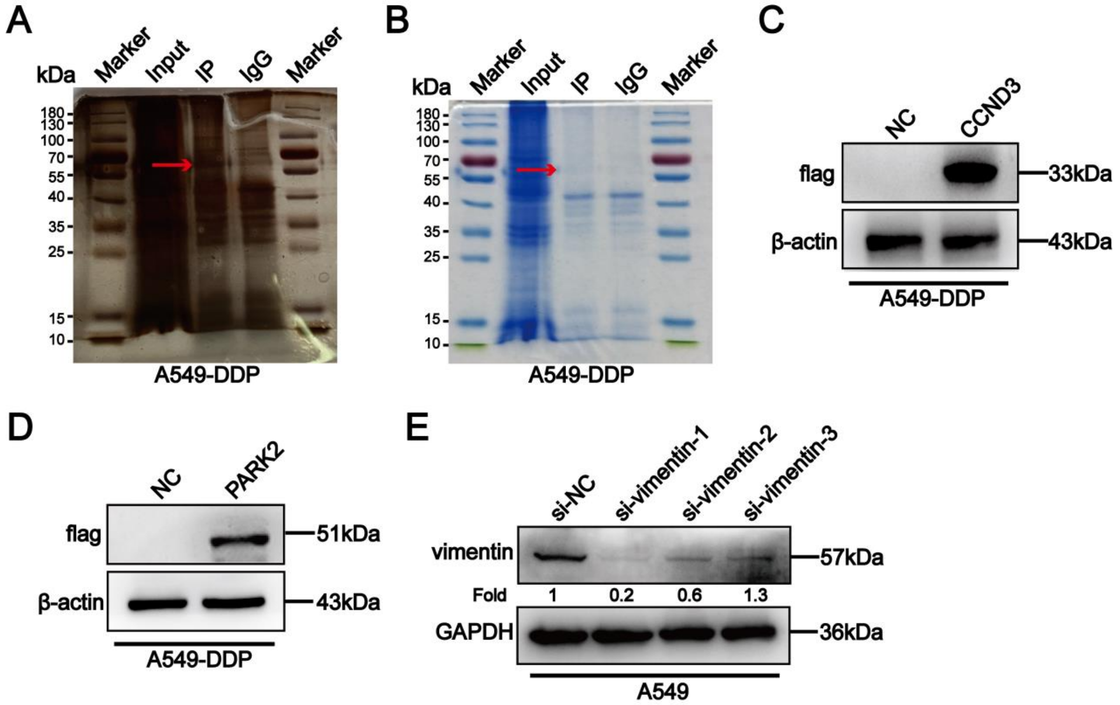

Fig. S4 Plasmids, siRNAs efficiency detection and protein staining. (A, and B) Silver staining (A) and Coomassie Brilliant Blue staining (B) on polyacrylamide gels following co-immunoprecipitation displayed the interacting protein bands of CCND3. Input served as a positive control. Red arrows represent the location of stained vimentin protein. (C) The overexpression of CCND3 in A549-DDP cells after transfection of the Flag-tagged CCND3 plasmids was evaluated by Western blot analysis. β-actin was used as a loading control. (D) The overexpression of PARK2 in A549-DDP cells after transfection of the Flag-tagged PARK2 plasmids was evaluated by Western blot analysis. β-actin was used as a loading control. (E) The knockdown efficiency of three siRNAs targeting vimentin in A549 cells was evaluated by Western blot analysis. GAPDH was used as a loading control.

Table S1.

Supplementary Table 1 The antibodies used in this study.

Antibody name	Brand name	Cat:	Dilution
β-actin	Fudebio-tech	FD0060	WB: 1:1000
β-Tubulin	CST	2128	WB: 1:1000
GAPDH	Proteintech Group	60004-1-AP	WB: 1:1000
CCND3	Proteintech Group	26755-1-AP	WB: 1:1000; IF: 1:50
CCND3	CST	2936	IHC: 1: 200
N-cadherin	CST	13116	WB: 1:1000
vimentin	CST	5741	WB: 1:1000
vimentin	Santa Cruz	sc-6260	IF: 1:50; IP: 1:70
snail	CST	3879	WB: 1:1000
E-cadherin	CST	3195	WB: 1:1000
p21	CST	2947	WB: 1:1000
p27	CST	3686	WB: 1:1000
CCND1	CST	2978	WB: 1:1000
CDK4	CST	12790	WB: 1:1000
NANOG	Proteintech Group	14295-1-AP	WB: 1:1000
Oct4	Proteintech Group	11263-1-AP	WB: 1:1000
PARK2	Santa Cruz	sc-32282	WB: 1:200; IF: 1:50
Ubiquitin	Proteintech Group	10201-2-AP	WB: 1:1000
c-Jun	CST	9165	WB: 1:1000; ChIP: 1:50
phospho-AKT	CST	4060	WB: 1:1000
phospho-PI3K	CST	17366	WB: 1:1000
flag	Sigma-Aldrich	F1804	WB: 1:1000; IP: 1:70
6×His	Proteintech Group	10001-0-AP	WB: 1:1000; IP: 1:70
HRP-conjugated Affinipure Goat Anti-Rabbit IgG (H+L)	Proteintech Group	SA00001-2	WB: 1:5000
HRP-conjugated Affinipure Goat Anti-Mouse IgG (H+L)	Proteintech Group	SA00001-1	WB: 1:5000
Goat anti-Mouse IgG (H+L) Cross-Adsorbed Secondary Antibody, Alexa Fluor™ 594	Invitrogen	A11005	IF: 1:200
Goat anti-Rabbit IgG (H+L) Cross-Adsorbed Secondary Antibody, Alexa Fluor™ 488	Invitrogen	A11008	IF: 1:500
Mouse IgG	CST	3420	IP: 1:70
Rabbit IgG	CST	2729	ChIP: 1:50

Table S2.

Supplementary Table 2 The target sequences of siRNAs used in this study.

mRNA name	NO.	Target Sequence
	1	ATCGCTACCTGTCTTGCGT
CCND3	2	AGATCGAAGCTGCACTCAG
	3	CTACAGATGTCACAGCCAT
	1	GCAGAAGAATGGTACAAAT
vimentin	2	CAACGAAACTTCTCAGCAT
	3	GGATGTTGACAATGCGTCT

Table S3.

Supplementary Table 3 The target sequences of shRNAs from the CCND3 mRNA sequence [GenBank: NM_001760].

NO.	Accession	Target Sequence	CDS	GC%
CCND3-RNAi (85145-3)	NM_001760	GCTGGTCCTAGGGAAGCTCAA	200..1078	57.14%
CCND3-RNAi (85146-11)	NM_001760	CTGCTGTGATTGCACATGATT	200..1078	42.86%
CCND3-RNAi (85147-1)	NM_001760	ATGGGACAGAATTGGATACAT	200..1078	38.10%
Description	Homo sapiens Cyclin D3 (CCND3), transcript variant 2, mRNA.			

Table S4.

Supplementary Table 4 The primers used in this study.

Primers name		Sequence (5’-3’)
GAPDH	Forward	GAACGGGAAGCTCACTGG
	Reverse	GCCTGCTTCACCACCTTCT
CCND3	Forward	ACCTGGCTGCTGTGATTGC
	Reverse	GATCATGGATGGCGGGTAC
c-Jun	Forward	AACTCGGACCTCCTCACCTC
	Reverse	TCATCTGTCACGTTCTTGGGG
promoter of CCND3 site 1	Forward	TCAGGTCAGGAGTTCGAGAC
	Reverse	GCCTCCCGGATTCAAGCTAT
promoter of CCND3 site 2	Forward	ATGGTACTTCGGGCACTTGA
	Reverse	ACTCTTACTACACGTCAGGCA

Table S5.

Supplementary Table 5 Correlation between CCND3 expression and the clinicopathological features of patients with lung adenocarcinomas (LUAD).

Variables	N	CCND3 expression		χ^2	p^* value
		low	high		
**Gender**					
**Male**	111	46(41.4%)	65(58.6%)		
**Female**	102	49(48.0%)	53(52.0%)	0.936	0.333
**Age**					
**≤50**	41	21(51.2%)	20(48.8%)		
**>50**	172	74(43.0%)	98(57.0%)	0.900	0.343
**Clinical stage**					
**I-II**	154	51(33.1%)	103(66.9%)		
**III-IV**	59	44(74.6%)	15(25.4%)	29.675	**0.000**
**T stage**					
**T1-T2**	175	78(44.6%)	97(55.4%)		
**T3-T4**	38	17(44.7%)	21(55.3%)	0.000	0.985
**N stage**					
**N0**	105	30(28.6%)	75(71.4%)		
**N1-N3**	108	65(60.2%)	43(39.8%)	21.535	**0.000**
**M stage**					
**M0**	205	88(42.9%)	117(57.1%)		
**M1**	8	7(87.5%)	1(12.5%)	4.519	**0.034**

[*] Chi-square test was applied to assess the association between CCND3 expression and the clinicopathological parameters.

Table S6.

Supplementary Table 6 The expression levels of CCND3 protein in primary LUAD tissues and metastatic LUAD tissues of lymph node.

Classification	N	CCND3 expression		p^* value
		low	high	
**Primary LUAD tissues**	213	95(44.6%)	118(55.4%)	
**Metastatic LUAD tissues of lymph node**	12	12(100.0%)	0(0.0%)	**0.000**

* Fisher's exact test was applied to assess the expression of CCND3 in the primary LUAD group and the metastatic LUAD group.

Table S7.

Supplementary Table 7 CCND3-binding proteins from the approximate 55-70 kDa band screened by liquid chromatography-tandem mass spectrometry.

	Accession	Score	Coverage (%)*	pI	Protein description
1	ZZZ3_HUMAN	3784	20.3	5.62	ZZ-type zinc finger-containing protein 3 OS=Homo sapiens GN=ZZZ3 PE=1 SV=1
2	ACTB_HUMAN	1224	57.1	5.29	Actin, cytoplasmic 1 OS=Homo sapiens GN=ACTB PE=1 SV=1
3	ALBU_HUMAN	973	19.2	5.92	Serum albumin OS=Homo sapiens GN=ALB PE=1 SV=2
4	MYH9_HUMAN	954	16.6	5.5	Myosin-9 OS=Homo sapiens GN=MYH9 PE=1 SV=4
5	ACTC_HUMAN	575	30.2	5.23	Actin, alpha cardiac muscle 1 OS=Homo sapiens GN=ACTC1 PE=1 SV=1
6	KPYM_HUMAN	534	38	7.96	Pyruvate kinase PKM OS=Homo sapiens GN=PKM PE=1 SV=4

7	ACTN4_HUMAN	530	24.9	5.27	Alpha-actinin-4 OS=Homo sapiens GN=ACTN4 PE=1 SV=2
8	POTEE_HUMAN	513	9.5	5.83	POTE ankyrin domain family member E OS=Homo sapiens GN=POTEE PE=1 SV=3
**9**	**VIME_HUMAN**	**480**	**39.7**	**5.06**	**Vimentin OS=Homo sapiens GN=VIM PE=1 SV=4**
10	CLH1_HUMAN	452	15.3	5.48	Clathrin heavy chain 1 OS=Homo sapiens GN=CLTC PE=1 SV=5
11	K2C1_HUMAN	425	17.9	8.15	Keratin, type II cytoskeletal 1 OS=Homo sapiens GN=KRT1 PE=1 SV=6
12	CH60_HUMAN	379	31.2	5.7	60 kDa heat shock protein, mitochondrial OS=Homo sapiens GN=HSPD1 PE=1 SV=2
13	ACTBL_HUMAN	339	17.6	5.39	Beta-actin-like protein 2 OS=Homo sapiens GN=ACTBL2 PE=1 SV=2
14	AL1A1_HUMAN	328	36.7	6.3	Retinal dehydrogenase 1 OS=Homo sapiens GN=ALDH1A1 PE=1 SV=2
15	TKT_HUMAN	256	25.8	7.58	Transketolase OS=Homo sapiens GN=TKT PE=1 SV=3
16	CAPZB_HUMAN	187	17.3	5.36	F-actin-capping protein subunit beta OS=Homo sapiens GN=CAPZB PE=1 SV=4
17	NUCL_HUMAN	176	10.6	4.6	Nucleolin OS=Homo sapiens GN=NCL PE=1 SV=3
18	MYH14_HUMAN	172	2	5.52	Myosin-14 OS=Homo sapiens GN=MYH14 PE=1 SV=2

19	TPM4_HUMAN	168	26.6	4.67	Tropomyosin alpha-4 chain OS=Homo sapiens GN=TPM4 PE=1 SV=3
20	K1C10_HUMAN	167	11.3	5.13	Keratin, type I cytoskeletal 10 OS=Homo sapiens GN=KRT10 PE=1 SV=6
21	ACTN2_HUMAN	163	5.6	5.31	Alpha-actinin-2 OS=Homo sapiens GN=ACTN2 PE=1 SV=1
22	MYH11_HUMAN	150	3.1	5.42	Myosin-11 OS=Homo sapiens GN=MYH11 PE=1 SV=3
23	AL3A1_HUMAN	149	10.8	6.11	Aldehyde dehydrogenase, dimeric NADP-preferring OS=Homo sapiens GN=ALDH3A1 PE=1 SV=3
24	K2C7_HUMAN	133	14.3	5.4	Keratin, type II cytoskeletal 7 OS=Homo sapiens GN=KRT7 PE=1 SV=5
25	K1C9_HUMAN	128	7.9	5.14	Keratin, type I cytoskeletal 9 OS=Homo sapiens GN=KRT9 PE=1 SV=3
26	K22E_HUMAN	116	4.9	8.07	Keratin, type II cytoskeletal 2 epidermal OS=Homo sapiens GN=KRT2 PE=1 SV=2
27	HSP7C_HUMAN	114	6.3	5.37	Heat shock cognate 71 kDa protein OS=Homo sapiens GN=HSPA8 PE=1 SV=1
28	MYL6_HUMAN	109	30.5	4.56	Myosin light polypeptide 6 OS=Homo sapiens GN=MYL6 PE=1 SV=2
29	CKAP4_HUMAN	106	11.6	5.63	Cytoskeleton-associated protein 4 OS=Homo sapiens GN=CKAP4 PE=1 SV=2
30	K2C5_HUMAN	103	5.1	7.59	Keratin, type II cytoskeletal 5 OS=Homo sapiens GN=KRT5 PE=1 SV=3

31	G6PI_HUMAN	101	9.9	8.43	Glucose-6-phosphate isomerase OS=Homo sapiens GN=GPI PE=1 SV=4
32	K2C1B_HUMAN	98	5	5.73	Keratin, type II cytoskeletal 1b OS=Homo sapiens GN=KRT77 PE=2 SV=3
33	LMNA_HUMAN	97	7.5	6.57	Prelamin-A/C OS=Homo sapiens GN=LMNA PE=1 SV=1
34	K2C6B_HUMAN	96	6.7	8.09	Keratin, type II cytoskeletal 6B OS=Homo sapiens GN=KRT6B PE=1 SV=5
35	ANXA2_HUMAN	95	14.2	7.57	Annexin A2 OS=Homo sapiens GN=ANXA2 PE=1 SV=2
36	MYH10_HUMAN	92	2.8	5.44	Myosin-10 OS=Homo sapiens GN=MYH10 PE=1 SV=3
37	GRP75_HUMAN	90	5.2	5.87	Stress-70 protein, mitochondrial OS=Homo sapiens GN=HSPA9 PE=1 SV=2
38	TBB5_HUMAN	86	4.7	4.78	Tubulin beta chain OS=Homo sapiens GN=TUBB PE=1 SV=2
39	EF1A1_HUMAN	84	6.7	9.1	Elongation factor 1-alpha 1 OS=Homo sapiens GN=EEF1A1 PE=1 SV=1
40	MYO1C_HUMAN	80	5.3	9.46	Unconventional myosin-Ic OS=Homo sapiens GN=MYO1C PE=1 SV=4
41	GSTP1_HUMAN	79	36.2	5.43	Glutathione S-transferase P OS=Homo sapiens GN=GSTP1 PE=1 SV=2
42	TCPQ_HUMAN	73	7.8	5.42	T-complex protein 1 subunit theta OS=Homo sapiens GN=CCT8 PE=1 SV=4

43	UGDH_HUMAN	68	4.7	6.73	UDP-glucose 6-dehydrogenase OS=Homo sapiens GN=UGDH PE=1 SV=1
44	ATPA_HUMAN	66	6.7	9.16	ATP synthase subunit alpha, mitochondrial OS=Homo sapiens GN=ATP5A1 PE=1 SV=1
45	K2C3_HUMAN	61	3	6.12	Keratin, type II cytoskeletal 3 OS=Homo sapiens GN=KRT3 PE=1 SV=3
46	MYL1_HUMAN	61	8.2	4.97	Myosin light chain 1/3, skeletal muscle isoform OS=Homo sapiens GN=MYL1 PE=1 SV=3
47	AL1L1_HUMAN	57	1.2	5.63	Cytosolic 10-formyltetrahydrofolate dehydrogenase OS=Homo sapiens GN=ALDH1L1 PE=1 SV=2
48	K1C14_HUMAN	56	8.3	5.09	Keratin, type I cytoskeletal 14 OS=Homo sapiens GN=KRT14 PE=1 SV=4
49	TCPH_HUMAN	56	6.8	7.55	T-complex protein 1 subunit eta OS=Homo sapiens GN=CCT7 PE=1 SV=2
50	PSMD3_HUMAN	55	1.7	8.47	26S proteasome non-ATPase regulatory subunit 3 OS=Homo sapiens GN=PSMD3 PE=1 SV=2
51	TCPZ_HUMAN	55	6	6.23	T-complex protein 1 subunit zeta OS=Homo sapiens GN=CCT6A PE=1 SV=3
52	ENOA_HUMAN	55	7.8	7.01	Alpha-enolase OS=Homo sapiens GN=ENO1 PE=1 SV=2
53	RO52_HUMAN	54	1.9	5.98	E3 ubiquitin-protein ligase TRIM21 OS=Homo sapiens GN=TRIM21 PE=1 SV=1

54	TCPG_HUMAN	53	4	6.1	T-complex protein 1 subunit gamma OS=Homo sapiens GN=CCT3 PE=1 SV=4
55	TPM3_HUMAN	52	17.5	4.68	Tropomyosin alpha-3 chain OS=Homo sapiens GN=TPM3 PE=1 SV=2
56	TBA1A_HUMAN	51	8.6	4.94	Tubulin alpha-1A chain OS=Homo sapiens GN=TUBA1A PE=1 SV=1
57	CLH2_HUMAN	50	3.1	5.57	Clathrin heavy chain 2 OS=Homo sapiens GN=CLTCL1 PE=1 SV=2
58	PUF60_HUMAN	47	7.5	5.19	Poly(U)-binding-splicing factor PUF60 OS=Homo sapiens GN=PUF60 PE=1 SV=1
59	TCPA_HUMAN	46	3.2	5.8	T-complex protein 1 subunit alpha OS=Homo sapiens GN=TCP1 PE=1 SV=1
60	1433B_HUMAN	45	3.3	4.76	14-3-3 protein beta/alpha OS=Homo sapiens GN=YWHAB PE=1 SV=3
61	WDR1_HUMAN	44	5.1	6.17	WD repeat-containing protein 1 OS=Homo sapiens GN=WDR1 PE=1 SV=4
62	MCLN2_HUMAN	42	1.2	7.73	Mucolipin-2 OS=Homo sapiens GN=MCOLN2 PE=2 SV=2
63	RL12_HUMAN	39	5.5	9.48	60S ribosomal protein L12 OS=Homo sapiens GN=RPL12 PE=1 SV=1
64	AL8A1_HUMAN	38	1.6	6.76	Aldehyde dehydrogenase family 8 member A1 OS=Homo sapiens GN=ALDH8A1 PE=1 SV=1
65	TPM1_HUMAN	36	13.7	4.69	Tropomyosin alpha-1 chain OS=Homo sapiens GN=TPM1 PE=1 SV=2

66	AMYP_HUMAN	35	2	6.6	Pancreatic alpha-amylase OS=Homo sapiens GN=AMY2A PE=1 SV=2
67	HSP71_HUMAN	33	4.4	5.48	Heat shock 70 kDa protein 1A/1B OS=Homo sapiens GN=HSPA1A PE=1 SV=5
68	PABP1_HUMAN	33	1.3	9.52	Polyadenylate-binding protein 1 OS=Homo sapiens GN=PABPC1 PE=1 SV=2
69	UACA_HUMAN	31	0.5	6.6	Uveal autoantigen with coiled-coil domains and ankyrin repeats OS=Homo sapiens GN=UACA PE=1 SV=2
70	DYH17_HUMAN	31	0.2	5.56	Dynein heavy chain 17, axonemal OS=Homo sapiens GN=DNAH17 PE=1 SV=2
71	AP2B1_HUMAN	31	1.1	5.22	AP-2 complex subunit beta OS=Homo sapiens GN=AP2B1 PE=1 SV=1
72	IGHG1_HUMAN	30	3.6	8.46	Ig gamma-1 chain C region OS=Homo sapiens GN=IGHG1 PE=1 SV=1
73	K2C71_HUMAN	30	5.7	6.28	Keratin, type II cytoskeletal 71 OS=Homo sapiens GN=KRT71 PE=1 SV=3
74	KRT82_HUMAN	30	1.4	6.4	Keratin, type II cuticular Hb2 OS=Homo sapiens GN=KRT82 PE=1 SV=3
75	ARP5_HUMAN	30	1.5	5.17	Actin-related protein 5 OS=Homo sapiens GN=ACTR5 PE=1 SV=2
76	G3P_HUMAN	29	7.8	8.57	Glyceraldehyde-3-phosphate dehydrogenase OS=Homo sapiens GN=GAPDH PE=1 SV=3

77	COR1C_HUMAN	29	5.3	6.65	Coronin-1C OS=Homo sapiens GN=CORO1C PE=1 SV=1
78	NFM_HUMAN	28	3.2	4.9	Neurofilament medium polypeptide OS=Homo sapiens GN=NEFM PE=1 SV=3
79	XPP3_HUMAN	28	1.4	6.37	Probable Xaa-Pro aminopeptidase 3 OS=Homo sapiens GN=XPNPEP3 PE=1 SV=1
80	HS90A_HUMAN	28	1	4.94	Heat shock protein HSP 90-alpha OS=Homo sapiens GN=HSP90AA1 PE=1 SV=5
81	AXDN1_HUMAN	27	1.5	5.49	Axonemal dynein light chain domain-containing protein 1 OS=Homo sapiens GN=AXDND1 PE=2 SV=1
82	RPN2_HUMAN	27	1.4	5.44	Dolichyl-diphosphooligosaccharide--protein glycosyltransferase subunit 2 OS=Homo sapiens GN=RPN2 PE=1 SV=3
83	STRN4_HUMAN	26	1.2	5.21	Striatin-4 OS=Homo sapiens GN=STRN4 PE=1 SV=2
84	PCLO_HUMAN	26	0.4	6.09	Protein piccolo OS=Homo sapiens GN=PCLO PE=1 SV=4
85	DYHC2_HUMAN	25	0.1	6.13	Cytoplasmic dynein 2 heavy chain 1 OS=Homo sapiens GN=DYNC2H1 PE=1 SV=4
86	NONO_HUMAN	25	2.5	9.01	Non-POU domain-containing octamer-binding protein OS=Homo sapiens GN=NONO PE=1 SV=4
87	AP2A1_HUMAN	24	2.6	6.63	AP-2 complex subunit alpha-1 OS=Homo sapiens GN=AP2A1 PE=1 SV=3

88	UBB_HUMAN	23	2.6	6.86	Polyubiquitin-B OS=Homo sapiens GN=UBB PE=1 SV=1
89	PCLI1_HUMAN	22	2.8	6.53	PTB-containing, cubilin and LRP1-interacting protein OS=Homo sapiens GN=PID1 PE=1 SV=1
90	ZZEF1_HUMAN	21	0.3	5.62	Zinc finger ZZ-type and EF-hand domain-containing protein 1 OS=Homo sapiens GN=ZZEF1 PE=1 SV=6
91	KV201_HUMAN	21	11.3	5.28	Ig kappa chain V-II region Cum OS=Homo sapiens PE=1 SV=1
92	TIGAR_HUMAN	20	2.6	7.6	Fructose-2,6-bisphosphatase TIGAR OS=Homo sapiens GN=TIGAR PE=1 SV=1
93	S12A7_HUMAN	16	0.7	6.28	Solute carrier family 12 member 7 OS=Homo sapiens GN=SLC12A7 PE=1 SV=3

* Percentage of the protein sequence covered by the matched peptides.
